# Effect of sarcopenia on treatment response and operative and oncological outcomes among patients undergoing neoadjuvant chemotherapy for breast cancer

**DOI:** 10.1093/bjsopen/zraf128

**Published:** 2025-11-04

**Authors:** Thomas O Butler, Jessie A Elliott, Matthew G Davey, Patrick M Collins, Megan McNamara, Eoin O'Malley, Micheal J Brennan, Kevin Barry, Sami Abd Elwahab, Karl Sweeney, Carmel Malone, Ray McLaughlin, Aoife Lowery, Michael J Kerin

**Affiliations:** Lambe Institute for Translational Research, Department of Surgery, University of Galway, Galway, Ireland; Lambe Institute for Translational Research, Department of Surgery, University of Galway, Galway, Ireland; Lambe Institute for Translational Research, Department of Surgery, University of Galway, Galway, Ireland; Department of Surgery, University Hospital Galway, Galway, Ireland; Department of Radiology, University Hospital Galway, Galway, Ireland; Department of Radiology, University Hospital Galway, Galway, Ireland; Department of Surgery, University Hospital Galway, Galway, Ireland; Department of Surgery, University Hospital Galway, Galway, Ireland; Department of Surgery, University Hospital Galway, Galway, Ireland; Department of Surgery, University Hospital Galway, Galway, Ireland; Department of Surgery, University Hospital Galway, Galway, Ireland; Department of Surgery, University Hospital Galway, Galway, Ireland; Lambe Institute for Translational Research, Department of Surgery, University of Galway, Galway, Ireland; Department of Surgery, University Hospital Galway, Galway, Ireland; Lambe Institute for Translational Research, Department of Surgery, University of Galway, Galway, Ireland; Department of Surgery, University Hospital Galway, Galway, Ireland

**Keywords:** survival, prognosis

## Abstract

**Background:**

Sarcopenia has been associated with adverse outcomes in numerous malignancies. The prevalence and prognostic significance of sarcopenia in patients with breast cancer receiving neoadjuvant chemotherapy (NAC) is uncertain. This study assessed the prevalence and effect of sarcopenia on the response to NAC, as well as on operative and oncological outcomes.

**Methods:**

Consecutive patients with breast cancer receiving NAC with curative intent between 2010 and 2015 at a single tertiary referral centre were included. Lean body mass and skeletal muscle index (SMI) were determined by analysing axial computed tomography scans taken at L3, with sarcopenia defined as SMI < 38.5cm^2^/m^2^. Univariable and multivariable linear, logistic, and Cox proportional hazards regression analyses were performed.

**Results:**

Among 258 patients (mean age 49.5 (SD11.1) years and BMI 27.6(5.7) kg/m^2^), 24 (12.2%) exhibited sarcopenia. Sarcopenia was not associated with molecular subtype (*P* = 0.746) nor clinical tumour size (*P* = 0.960). On multivariable analysis, sarcopenia did not predict complete pathological response (*P* = 0.069), nodal positivity after NAC (*P* = 0.442), or Sataloff tumour response to NAC (*P* = 0.898). Sarcopenia did not predict the length of hospital stay (*P* = 0.716) nor the Comprehensive Complication Index (*P* = 0.242) after surgery. Lower lean body mass independently predicted overall survival (hazard ratio (HR) 0.92; 95% confidence interval [c.i.] 0.85 to 0.99; *P* = 0.028) and invasive disease-free survival (HR 0.93; 95% c.i. 0.87 to 1.00; *P* = 0.049), but not disease-specific survival (*P* = 0.070).

**Conclusion:**

Sarcopenia was not associated with clinicopathological parameters and did not affect the response to NAC nor postoperative complications. Lower lean body mass was associated with reduced overall and invasive disease-free survival in patients with breast cancer receiving NAC.

## Teaser

The association between sarcopenia and clinicopathological characteristics and prognostic outcomes among patients receiving neoadjuvant chemotherapy for breast cancer is unclear. This study found that sarcopenia was not associated with poorer prognosis, but lower lean body mass was associated with reduced overall and invasive disease-free survival.

## Introduction

Breast cancer is a heterogeneous disease and is the most commonly diagnosed cancer in women^1^. Advances in tumour identification and treatment have led to improved oncological outcomes for patients with breast cancer; the current 5-year relative survival rates for patients diagnosed with stage 1 and stage 2 disease are >99 and 93%, respectively^2^. As a result, patients with breast cancer are now acknowledged to live with a significant burden of morbidity associated with existing treatment options, including the use of cytotoxic chemotherapy^3^. As such, a major focus of current research is to identify accurate biomarkers that can be used to personalize management, limit morbidity and the late effects of treatment, and optimize oncological outcomes^4^.

Neoadjuvant chemotherapy (NAC) has been shown to result in tumour downstaging, increased resectability, and enhanced eligibility for breast-conserving surgery^5^; it also provides information with respect to the *in vivo* sensitivity of the tumour to systemic therapies, which is now recognized to harbour prognostic value^6^. Following the administration of NAC, current evidence indicates that the presence of a major or complete pathological response (pCR) represents an important prognostic outcome among patients with breast cancer^6,7^, and the 2021 American Society of Clinical Oncology (ASCO) guidelines^8^ have consequently recommended using pCR to measure the response to NAC.

There is growing interest in the importance of nutritional factors in the management of patients being treated for breast cancer^9^. Weight loss may occur among patients with breast cancer for numerous reasons, including toxic adverse effects of chemotherapy and cancer-related cachexia^10^, whereas weight gain and increased adiposity can occur due to factors such as reduced physical activity secondary to cancer- and treatment-related fatigue^9,11^. However, changes in body weight may not reliably reflect changes in body composition^12^. Sarcopenia is characterized by a reduction in skeletal muscle mass and function^13^ and has a multifactorial aetiology; it is promoted by physical inactivity, systemic inflammation, increased metabolic rate, and malnutrition^14^. In oncology, the presence of sarcopenia can be assessed opportunistically using computed tomography (CT) images, which has driven research into its potential role as a clinical biomarker. Accordingly, the presence of sarcopenia has now been associated with adverse operative and oncological outcomes, as well as increased treatment toxicity, in many conditions, including lung, colorectal, gastric, pancreatic, and oesophageal cancer^14–16^.

Previous studies have investigated the prevalence of sarcopenia in patients with breast cancer^17–19^; however, these studies were limited by small sample sizes and used different methods of defining sarcopenia. The prognostic significance of sarcopenia in patients with breast cancer also remains uncertain^20^. Furthermore, although sarcopenia has been associated with poor response to NAC in other malignancies^14^, there is a paucity of studies exploring the prevalence of sarcopenia in patients who are receiving NAC for breast cancer and how this affects treatment response^21^. A recent meta-analysis^22^ of seven studies, including early-stage and metastatic disease, found that sarcopenia was associated with increased mortality risk among patients with breast cancer and highlighted a need to further elucidate the prognostic value of sarcopenia in patients with early-stage disease.

Thus, the aims of the present study were to determine the prevalence of sarcopenia in patients with breast cancer indicated to receive NAC in a high-volume European tertiary referral centre, and to establish how sarcopenia affects the response to NAC. In addition, this study aimed to investigate the impact of sarcopenia and body composition on operative and oncological outcomes.

## Methods

### Patient selection and study design

This study was approved by the Ethics Committee of University Hospital Galway (Approval No. C.A. 2377). A single-centre observational retrospective cohort study was performed at the Breast Cancer Centre at Galway University Hospital, which is a high-volume national centre, in the west of Ireland. The inclusion criteria were patients who received NAC with curative intent and surgery for breast cancer between 2010 and 2015; the exclusion criteria were patients treated with neoadjuvant endocrine therapy only and patients without adequate CT scans to assess body composition. Given the natural history of breast cancer, patients diagnosed from 2010 to 2015 were included to ensure 5 years of follow up for outcome data. Once eligibility criteria were applied, 258 patients were considered fit for inclusion. Detailed clinicopathological information was obtained from a comprehensive institutional database, which is prospectively maintained for each patient with breast cancer, as reported previously^23^.

### Clinical and histopathological assessment

Oestrogen receptor (ER) and progesterone receptor (PR) status was analysed routinely using the Allred scoring system^24^. Included in the histopathological assessment, human epidermal growth factor receptor 2 (HER2) receptor status was identified using HercepTest^TM^ kits (Agilent Technologies, Santa Clara, CA, USA), with a a score of 3+ considered positive^25^. Borderline HER2 results (that is, scores of 2+), underwent fluorescence *in situ* hybridization analysis for more detailed characterization, as per ASCO guidelines^26^.

When sentinel lymph node biopsy (SLNB) was performed before treatment, these samples were included when assessing the total nodal burden from surgical samples to determine the final pathological nodal status. For patients with synchronous bilateral or multifocal tumours, the node-positive primary tumour was included, if node positive. If node negative, the larger primary tumour was used for the purpose of this analysis.

### Treatment protocol

Patients were indicated to receive NAC if they had clinical node positivity, nodal metastases that were pathologically confirmed on SLNB, triple-negative or HER2-positive tumours, a primary tumour that was at least 5 cm in diameter or at the threshold for being suitable for breast conserving surgery^27^; all patients were discussed at the weekly breast cancer multidisciplinary meeting. Patients who underwent NAC in accordance with multidisciplinary tumour board recommendations were considered eligible for the present study. Patients included in this study were given Adriamycin^®^ (doxorubicin, Pfizer Inc, New York City, NY, USA) and cyclophosphamide before receiving paclitaxel (AC-T regimen, dose dense), where this was achievable^28^. Patients underwent resection 6 weeks after completing their course of NAC, and breast-conserving surgery was performed where feasible.

A multidisciplinary enhanced recovery protocol was implemented, with early upper limb mobilization supervised by a specialist breast physiotherapist, and dedicated breast care education conducted by clinical nurse specialists.

All ER- and PR-positive patients were given adjuvant endocrine therapy for a minimum of 5 years after surgery, as tolerated^29^. Adjuvant radiotherapy was indicated following breast-conserving surgery or for positive surgical margins, a primary tumour size ≥ 5 cm, post-neoadjuvant chemotherapy stage T4 disease and axillary nodal involvement of four or more nodes^30,31^. Patients were given the conventional regimen of 50 Gy in 25 fractions, as reported previously^32^, a higher dose of 50.4 Gy in 38 fractions, or a hypofractionation regimen of 40 Gy in 15 fractions when necessary^33^.

### Variable definitions

Response to NAC was graded using the Sataloff tumour and nodal scores^34^. A pCR meant that there were no viable tumour cells, either at the primary tumour site or within the nodal basin. The Clavien–Dindo system^35^ and Comprehensive Complications Index^36^ (CCI) were used to assess postoperative complications.

Local recurrence referred to disease that was limited to the ipsilateral chest wall on recurrence; regional recurrence referred to disease recurring in the axillary, supraclavicular, or internal mammary lymph nodes at first relapse. Distant recurrence was defined as the recurrence of disease in a site separate to these definitions. Invasive disease-free survival (iDFS) was defined as the absence of invasive disease recurrence, second primary cancer, or death. Overall survival (OS) was defined as the absence of death occurring regardless of disease recurrence.

### CT assessment of body composition

**Fig. 1:**
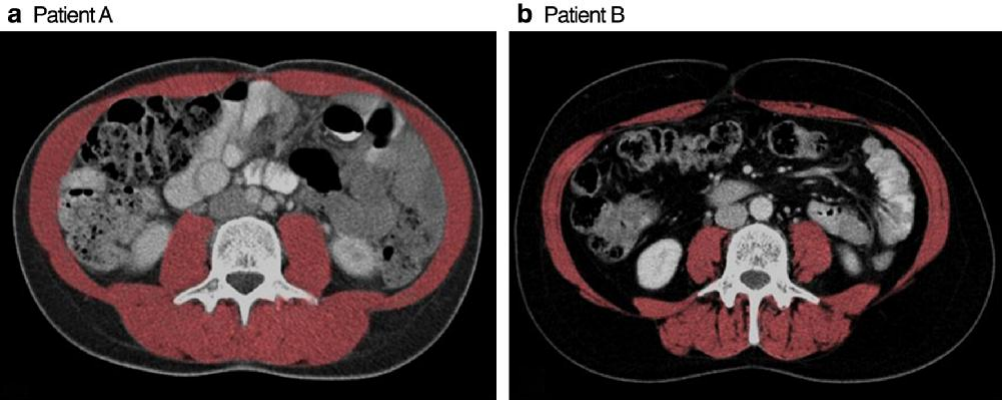
Computed tomography assessment of body composition Abdominal computed tomography for two patients with breast cancer. **a** Patient A had a BMI of 22.8 kg/m^2^; **b** Patient B had a BMI of 23.7 kg/m^2^. Despite similar BMIs, Patient A has normal lean tissue and visceral fat areas, whereas Patient B has both visceral obesity and sarcopenia. BMI, body mass index.

Axial CT scans were performed at diagnosis using a multislice Somatom Sensation scanner (Siemens Healthcare, Erlangen, Germany) as part of routine imaging. Single-blinded investigators used OsiriX MD (Pixmeo SARL, Geneva, Switzerland) to analyse images taken at L3 and determine the cross-sectional area of separate tissue compartments. A semiautomated algorithm was applied that used ranges of −29 to 150 Hounsfield units (HU) for skeletal muscle and −50 to −150 HU for adipose tissue on CT images^37–39^ (*Fig. 1*).

The skeletal muscle index (SMI) was calculated as the ratio of lean tissue area to height using the following formula:


$$SMI=\frac{Lean\;tissue\;area\;at\;L3\;(cm^{2})}{Height\;(m^{2})}$$

Sarcopenia was defined as an SMI < 38.5 cm^2^/m^2 37,39^. To numerically quantify the amount of skeletal muscle present in patients, lean body mass (LBM) was calculated using the following formula, which was developed and validated against dual energy X-ray absorptiometry as the standard^37,39^:


$$LBM\;(kg)=0.30\times Lean\;tissue\;area\;at\;L3\;(cm^{2})+6.06$$

### Statistical analysis

Data were analysed using GraphPad Prism (v.6.0) for Windows (GraphPad Software, San Diego, CA, USA) and SPSS^®^ (v.23.0) software (IBM, Armonk, NY, USA). Clinicopathological and immunohistochemistry correlates of sarcopenia status and LBM were determined using independent Student's *t* test, the χ^2^ test, Fischer’s exact test, the Fisher–Freeman–Halton test, or one-way analysis of variance (ANOVA), as needed. To perform multivariable analyses, clinically relevant variables were inserted into linear and logistic regression analyses using a forward stepwise selection procedure. Kaplan–Meier curves were analysed using the log-rank test, and Cox proportional hazards regression models were used for multivariable survival analyses using a forward stepwise selection procedure. The threshold of significance was set at two-sided *P* < 0.050.

## Results

### Study population

In all, 345 patients received NAC for breast cancer. Of these patients, 258 had sufficient CT scans to assess body composition and represent the final study cohort (*Fig. S1*). Clinicopathological characteristics of the study population are presented in *Table 1*. The mean(standard deviation (s.d.)) age was 49.5(11.1) years and half the patients were premenopausal (134, 51.9%). A minority of tumours were screen-detected (17, 6.6%), most were invasive ductal carcinoma (189, 73.3%), most commonly ER positive (161, 62.4%), and over half were PR positive (141, 54.7%). In all, 127 tumours (49.2%) were luminal A, 45 (17.4%) were luminal B, 29 (11.2%) were HER2 positive, and 57 (22.1%) were basal-like tumours. Eighty-four patients (74.3%) with luminal A tumours were clinically node positive, 25 patients (66%) with luminal B tumours were clinically node positive, and 43 patients (57%) with other subtypes were clinically node positive.

**Table 1:** Clinicopathological and treatment characteristics and outcomes for the study population

**Clinical characteristics**		**Treatment characteristics**	
Age at diagnosis (years), mean(s.d.)	49.5(11.1)	Chemotherapy regimen	
Menopausal status		AC-T	155 (63.3%)
Premenopausal	134 (51.9%)	Other	90 (36.7%)
Perimenopausal	22 (8.5%)	Neoadjuvant HER2-directed treatment	65 (25.6%)
Postmenopausal	102 (39.5%)	Adjuvant radiation	229 (89.8%)
Screen detected	17 (6.6%)	Breast procedure*	
Genetic risk		Wide local excision	121 (46.9%)
*BRCA1*	6 (2.3%)	Mastectomy	137 (53.1%)
*BRCA2*	2 (0.8%)	Axillary procedure†	
**Pathological characteristics**		Sentinel node biopsy	60 (23.3%)
Affected breast		Axillary clearance	198 (76.1%)
Right	132 (51.2%)	Reconstruction	34 (29.8%)
Left	136 (48.8%)	Implant	24 (30%)
Histological type		Autologous	55 (70%)
Lobular	21 (8.1%)	**Postoperative outcomes**	
Other	48 (18.6%)	Any complication	35 (15.0%)
Background DCIS	139 (70.9%)	CCI, mean(s.d.)	3.1(8.7)
Tumour grade		Clavien–Dindo grade	
Grade 1	6 (2.4%)	No complication	198 (85.0%)
Grade 2	121 (48.0%)	Grade I	14 (6.0%)
Grade 3	125 (49.6%)	Grade II	12 (5.2%)
Receptor status		Grade III	
ER positive	161 (62.4%)	Grade IIIa	0 (0.0%)
ER Allred score, mean(s.d.)	4.5(3.6)	Grade IIIb	9 (3.9%)
PR positive	141 (54.7%)	Grade IV	0 (0.0%)
PR Allred score, mean(s.d.)	3.3(3.3)	Wound infection	17 (7.3%)
HER2 positive	73 (28.3%)	Haematoma	6 (2.6%)
Molecular subtype		Seroma	19 (8.2%)
Luminal A	127 (49.2%)	Delayed wound healing	2 (0.9%)
Luminal B	45 (17.4%)	Reoperation	9 (3.9%)
HER2	29 (11.2%)	Inpatient LOS (days), median (interquartile range)	6.0 (3.0–7.0)
Basal-like	57 (22.1%)	30-day readmission	5 (2.1%)
Multifocal disease	26 (10.1%)	In-hospital mortality	0 (0.0%)
Clinical stage		**Pathological response evaluation**	
T0	1 (0.4%)	Sataloff grade—primary tumour	
T1	31 (12.8%)	A	67 (33.2%)
T2	165 (67.9%)	B	75 (37.1%)
T3	36 (14.8%)	C	54 (26.7%)
T4	10 (4.1%)	D	6 (3.0%)
N0	74 (32.7%)	Sataloff grade—nodal	
N1–3	152 (67.2%)	A	40 (11.7%)
Pathological stage		B	61 (30.3%)
T0	54 (23.1%)	C	69 (34.4%)
T1	56 (23.9%)	D	31 (15.4%)
T2	72 (30.8%)	Pathological complete response	51 (19.8%)
T3	44 (18.8%)	Downstaged	120 (57.7%)
T4	8 (3.4%)		
N0	105 (49.5%)		
N1	57 (26.9%)		
N2	31 (14.6%)		
N3	19 (9.0%)		
No. of positive nodes, mean(s.d.)	3.3(5.6)		
Nodal yield, mean(s.d.)	14.3(8.8)		
Nottingham prognostic index			
1	4 (2.0%)		
2	4 (2.0%)		
3	131 (63.9%)		
4	66 (32.3%)		
R0 resection*	211 (98.6%)		

Values are *n* (%) unless otherwise stated. s.d., standard deviation; *BRCA1*, BRCA1 DNA repair associated; *BRCA2*, BRCA2 DNA repair associated; DCIS, ductal carcinoma *in situ*; ER, eostrogen receptor; PR, progesterone receptor; HER2, human epidermal growth factor receptor 2; AC-T, doxorubicin and cyclophosphamide followed by paclitaxel; CCI, Comprehensive Complication Index. LOS, length of hospital stay. *Final histological margin status (incorporates re-excision, if needed). †Final procedure.

### Treatment characteristics

The most commonly used NAC regimen was AC-T (155, 63.3%), after which mastectomy was performed in just over half the patients (137, 53.1%); an axillary clearance procedure was performed in 198 patients (76.1%). Reconstruction was performed in 34 patients (29.8%), which was predominantly autologous (55, 70%). Complications occurred in 35 patients (15.0%), with a mean(s.d.) CCI of 3.1(8.7). The Clavien–Dindo grade predominantly classified as no complication (198, 85.0%), with seroma being the most common complication (19, 8.2%). The median inpatient length of stay was 6.0 days (interquartile range 3.0–7.0 days). Further treatment characteristics are presented in *Table 1*.

### Body composition

Body composition parameters are presented in *Table 2*. Overall, 24 patients (12.2%) exhibited sarcopenia; the mean(standard deviation) BMI was 27.6(5.7) kg/m^2^ and mean LBM was 41.6(4.8) kg. Among the study cohort, 125 patients (74.8%) were in either the normal weight or overweight BMI category. Patients with sarcopenia were significantly less likely to exhibit visceral obesity than those without sarcopenia (25.0 *versus* 48.6%; *P* = 0.030; *Table S1*). Sarcopenia was less common among patients with a higher BMI (*P* = 0.001), being exhibited by 14 (23%), 5 (8%), and 0 (0%) patients with normal weight, overweight, and obesity, respectively. On average, sarcopenic patients were lighter than patients without sarcopenia (mean(standard deviation) 63.3(7.9) *versus* 74.1(15.1) kg; *P* < 0.001), but sarcopenia was not associated with percentage body weight loss at diagnosis, which was generally low (mean(standard deviation) −1.0%(6.7) *versus* −0.1%(5.0), respectively; *P* = 0.556).

**Table 2:** Body anthropometry and body composition

**Standard anthropometry**	
Weight (kg)	
Mean(s.d.)	72.4(14.8)
Range	42.9–147.8
Height (cm)	
Mean(s.d.)	162.0(5.7)
Range	146.7–176.0
BMI (kg/m^2^)	
Mean(s.d.)	27.6(5.7)
Range	17.6–56.9
BMI category	
Normal weight	61 (36.5%)
Overweight	64 (38.3%)
Obese	42 (25.1%)
**Body composition**	
Lean tissue area (cm^2^)	
Mean(s.d.)	118.4(16.0)
Range	76.0–173.7
Lean body mass, kg)	
Mean(s.d.)	41.6(4.8)
Range	28.9–58.2
Skeletal muscle index (cm^2^/m^2^)	
Mean(s.d.)	45.4(6.3)
Range	31.7–68.9
Sarcopenia	24 (12.2%)

Values are *n* (%) unless otherwise stated. s.d., standard deviation; BMI, body mass index.

### Response to NAC

Following NAC, pCR was achieved in 51 patients (19.8%), with a favourable Sataloff tumour response (grade A or B) occurring in 142 patients (70.3%). Six patients (3.0%) had a Sataloff tumour score of D. In all, 114 patients (53.8%) had pathologically negative nodes, with 40 patients (11.7%) exhibiting Sataloff nodal grade A.

### Oncological outcomes

The median(standard error) follow-up time was 79.1(3.2) months. Median OS and disease-specific survival (DSS) were not reached; the median iDFS was 74.2(4.4) months. The 3-year and 5-year DSS was 90.3(1.9)% and 82.1(2.6)%, respectively; the 3-year and 5-year OS was 88.4(2.0)% and 79.1(2.7)%, respectively; and the 3-year and 5-year iDFS was 81.3(2.5)% and 71.7(3.0)%, respectively.

### Sarcopenia and clinicopathological and treatment characteristics

Sarcopenia was not associated with baseline tumour stage, grade, or molecular subtype, but lower LBM was associated with postmenopausal status (*P* = 0.048; *Tables S2,S3*). Similarly, treatment strategies were similar among patients with and without sarcopenia, including the choice of neoadjuvant therapy, surgical procedure, and adjuvant therapies (*Table S4*).

On univariable analysis, sarcopenia was not associated with histopathological response variables, including ypT and ypN stage, pCR, or Sataloff tumour and nodal score (*P* = 0.475, *P* = 0.904, *P* = 0.087, *P* = 1.000, and *P* = 0.523, respectively; *Table 3*). Similarly, on multivariable analysis, pCR, post-neoadjuvant pathological nodal positivity (ypN+), and Sataloff tumour response were not associated with sarcopenia status (*Table 4*). However, greater LBM was independently associated with ypN+ (odds ratio 1.12; 95% confidence interval (c.i.) 1.02 to 1.24; *P* = 0.019), but not Sataloff tumour response or pCR.

**Table 3:** Association between sarcopenia and body composition, pathological response and postoperative outcome parameters

	No sarcopenia (*n* = 173)	Sarcopenia (*n* = 24)	*P*	LBM** (kg), mean(s.d.)	*P*
**Pathological response evaluation**					
Sataloff tumour score			1.000*		0.199†
A	41 (30.1%)	5 (29%)		41.8(4.9)	
B	54 (39.7%)	7 (41%)		41.1(4.0)	
C	38 (27.9%)	5 (29%)		42.6(5.3)	
D	3 (2.2%)	0 (0%)		39.3(4.3)	
Sataloff nodal score			0.523*		0.549†
A	24 (17.5%)	4 (24%)		42.1(5.0)	
B	43 (31.4%)	4 (24%)		41.8(4.6)	
C	47 (34.3%)	8 (47%)		41.0(4.4)	
D	23 (16.8%)	1 (6%)		42.2(5.1)	
pCR	32 (18.5%)	1 (4%)	0.087‡	42.3(4.5)/41.4(4.9)	0.257§
Post-NAC stage			0.475*		
0	30 (21.1%)	2 (11%)			
1a	23 (16.2%)	3 (17%)			
1b	1 (0.7%)	0 (0%)			
2a	25 (17.6%)	6 (33%)			
2b	15 (10.6%)	4 (22%)			
3a	33 (23.2%)	2 (11%)			
3b	2 (1.4%)	0 (0%)			
3c	13 (9.2%)	1 (6%)			
ypN status			0.904¶		0.885§
ypN1–3	81 (57.0%)	10 (56%)		41.6(5.0)	
ypN0	61 (43.0%)	8 (44%)		41.7(4.9)	
**Postoperative outcomes**					
Complications occurred	27 (16.8%)	2 (9%)	0.536‡	42.7(4.9)/41.5(4.8)	0.168§
Clavien–Dindo grade			0.943*		0.203†
No complication	134 (83.2%)	20 (91%)		41.5(4.8)	
Grade I	11 (6.8%)	1 (5%)		42.0(5.2)	
Grade II	8 (5.0%)	1 (5%)		41.8(5.6)	
Grade III					
Grade IIIa	0 (0%)	0 (0%)		0(0)	
Grade IIIb	8 (5.0%)	0 (0%)		45.0(2.7)	
Grade IV	0 (0%)	0 (0%)		0(0)	
Readmission within 30 days	5 (3.1%)	0 (0%)	1.000‡	43.5(2.3)/41.6(4.9)	0.396§
Wound infection	13 (8.1%)	1 (5%)	1.000‡	42.6(4.8)/41.6(4.9)	0.412§
Seroma	16 (9.9%)	1 (5%)	0.698‡	42.7(5.1)/41.6(4.8)	0.327§
Haematoma	5 (3.1%)	0 (0%)	1.000‡	45.3(3.1)/41.6(4.9)	0.063§
Delayed wound healing	2 (1.2%)	0 (0%)	1.000‡	42.0(2.9)/41.7(4.9)	0.920§
Reoperation	5 (3.1%)	0 (0%)	1.000‡	43.5(2.3)/41.6(4.9)	0.396§
Length of hospital stay (days), mean(s.d.)	5.4(3.8)	6.4(5.5)	0.248§		0.755#
CCI, mean(s.d.)	3.6(9.5)	1.3(4.7)	0.079§		0.068#

Values are *n* (%) unless indicated otherwise stated. **All LBM values are given as the mean(SD); values after the solidus denote comparison mean(s.d.) values used to calculate *P* values. LBM, lean body mass; s.d., standard deviation; pCR, pathological complete response; NAC, neoadjuvant chemotherapy; ypN1–3e, summative pathological node positivity after neoadjuvant chemotherapy; ypN0, summative pathological node negativity after neoadjuvant chemotherapy; CCI, comprehensive complication index. *Fisher-Freeman-Halton test, except †one-way analysis of variance, ‡Fisher's exact test, §Student’s independent *t* test, ¶ χ2 test, and #Pearson's correlation coefficient test.

**Table 4:** Multivariable analysis of factors associated with response to chemotherapy for breast cancer

	pCR		ypN+		Sataloff tumour response†	
	OR*	*P*	OR*	*P*	OR*	*P*
**Clinicopathological characteristics**						
Age		0.734		0.536		0.269
Menopausal status		0.642		0.689		0.405
BreastCheck detected		0.482		0.276		0.264
BRCA association		0.823		0.361		0.166
Tumour multifocality		0.576		0.893		0.785
Clinical tumour size		0.103		0.386		0.335
Clinical tumour stage (cT2–4 *versus* cT1)		0.460		0.814		0.308
Clinical nodal stage (cN1–3 *versus* cN0)		0.598	21.82 (6.83, 69.68)	<0.001		0.174
Tumour grade (G3 *versus* G1–2)		0.675		0.118		0.218
Histological subtype‡		0.493		0.290		0.495
ER status		0.870		0.649		0.104
ER Allred score		0.734		0.579		0.649
PR status		0.504		0.919		0.956
PR Allred score	0.77 (0.66, 0.91)	0.002	1.45 (1.23, 1.70)	<0.001		0.595
HER2 positivity		0.502		0.328		0.725
Breast Cancer receptor type**		0.676		0.399		0.007
Luminal B *versus* luminal A					0.24 (0.07, 0.78)	0.017
HER2 *versus* luminal A					0.16 (0.03, 0.73)	0.018
Basal *versus* luminal A					0.34 (0.13, 0.90)	0.030
**Treatment characteristics**						
Chemotherapy regimen (other *versus* AC-T)		0.484		0.477		0.895
Radiotherapy cycles completed		0.583				
Neoadjuvant HER2-directed treatment	4.24 (1.73, 10.37)	0.002	0.15 (0.05, 0.39)	<0.001		0.559
**Body composition**						
Sarcopenia		0.069		0.442		0.898
Lean body mass		0.959	1.12 (1.02, 1.24)	0.019		0.149

^*^Values in parentheses are 95% confidence intervals. **Defined as Luminal A, Luminal B, Her2 or basal. †Analysed as a categorical variable (ductal, lobular, other), category *P* values and ORs not significant on logistic regression. ‡Analysed as Sataloff A–B *versus* C–D. pCR, pathologic complete response; ypN+, summative pathological node positivity after neoadjuvant chemotherapy; OR, odds ratio; BRCA, breast cancer gene; ER, oestrogen receptor; PR, progesterone receptor; HER2, human epidermal growth factor receptor 2; AC-T, doxorubicin and cyclophosphamide followed by paclitaxel.

There was no significant relationship between the presence of sarcopenia or reduced LBM and the incidence of postoperative complications on univariable or multivariable analysis (*Table 3*; *Table S5*).

### Sarcopenia and oncological outcome

**Fig. 2:**
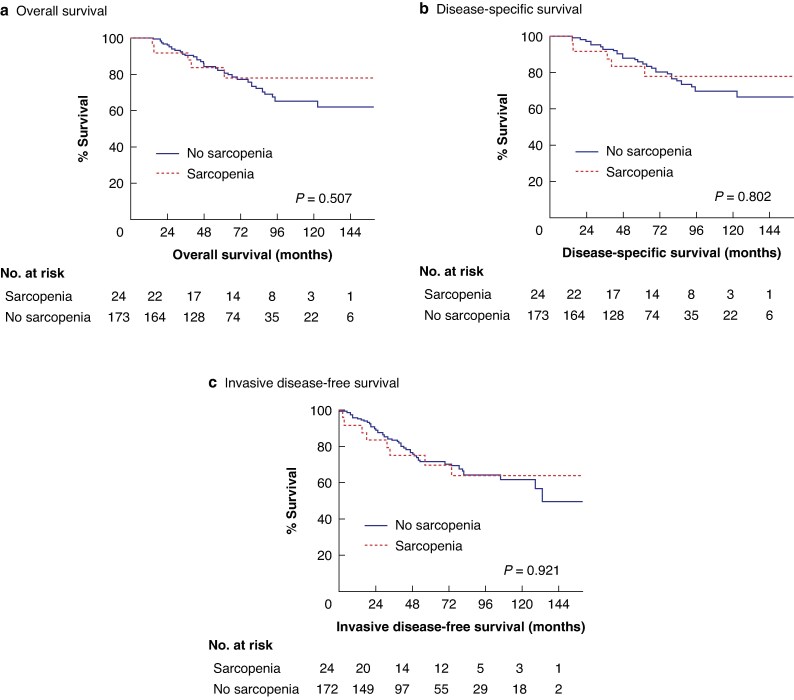
Sarcopenia and survival outcomes **a** Overall survival; **b** disease-specific survival; and **c** invasive disease-free survival.

On univariable analysis, sarcopenia had no significant effect on OS, DSS, or iDFS (*P* = 0.507, *P* = 0.802, and *P* = 0.921, respectively; *Fig. 2*). However, on multivariable analysis, lower LBM independently predicted reduced iDFS (hazard ratio (HR) 0.93; 95% c.i. 0.87 to 1.00; *P* = 0.049) and OS (HR 0.92; 95% c.i. 0.85 to 0.99; *P* = 0.028), but not DSS (*P* = 0.070), as indicated in *Table 5* and *Fig. S3*.

**Table 5:** Multivariable analysis of factors associated with oncological outcome after NAC for breast cancer

	iDFS		DSS		OS	
	HR*	*P*	HR*	*P*	HR*	*P*
**Clinicopathological characteristics**						
Age		0.392		0.433	1.04 (1.01, 1.07)	0.019
Menopausal status		0.957		0.745		0.991
BreastCheck detected		0.094		0.120		0.056
BRCA association		0.158		0.262		0.243
Tumour multifocality		0.818		0.249		0.284
Clinical tumour size	1.02 (1.01, 1.04)	0.004		0.076		0.834
Clinical tumour stage†		0.278		0.074		0.009
T2 *versus* T0–T1					1.60 (0.36, 7.05)	0.534
T3–T4 *versus* T0–T1					5.16 (1.07, 24.90)	0.041
Clinical nodal stage (cN1–3 *versus* cN0)		0.351		0.374		0.396
Pathological tumour size		0.057		0.477		0.375
Pathological nodal stage		<0.001		0.385		0.122
N1 *versus* N0	4.40 (1.93, 10.04)	<0.001				
N2 *versus* N0	6.53 (2.65, 16.11)	<0.001				
N3 *versus* N0	6.66 (2.52, 17.57)	<0.001				
Tumour grade (G3 *versus* G1–2)	3.15 (1.66, 5.98)	<0.001		0.057	2.95 (1.20, 6.21)	0.004
ER status		0.975		0.286		0.693
ER Allred score		0.509		0.215		0.362
PR status		0.924		0.776		0.959
PR Allred score		0.958		0.834		0.706
HER2 positivity		0.643		0.505		0.292
Breast Cancer receptor type**		0.281		0.457		0.206
Histological subtype†		0.780		0.957		0.672
R0 resection		0.651		0.356		0.736
**Treatment characteristics**						
NAC regimen (AC-T *versus* others)		0.427		0.849		0.736
Radiotherapy cycles complete		0.950		0.512		0.967
Adjuvant chemotherapy						
Adjuvant radiotherapy		0.079		0.517		0.201
Adjuvant hormonal therapy		0.922		0.949		0.864
Neoadjuvant HER2-directed treatment		0.883		0.836		0.771
Reconstruction performed		0.881		0.691		0.419
**Treatment response characteristics**						
pCR		0.137		0.941	0.92 (0.85, 0.99)	0.028
ypT stage†		0.837		0.657		0.538
Total positive node count		0.482	1.08 (1.03, 1.13)	0.002	1.06 (1.01, 1.11)	0.019
Downstaging		0.709		0.769		0.370
Complication occurrence		0.803		0.238		0.483
CCI		0.509		0.308		0.899
**Body composition**						
Lean body mass	0.93 (0.87, 1.00)	0.049		0.070	0.92 (0.85, 0.99)	0.028
Visceral obesity		0.822		0.759		0.578

^*^Values in parentheses are 95% confidence intervals. **Defined as Luminal A, Luminal B, Her2 or Basal. †Analysed as a categorical variable, category *P* values and HRs not significant on Cox proportional hazards regression. NAC, neoadjuvant chemotherapy; iDFS, invasive disease-free survival; DSS, disease-specific survival; OS, overall survival; HR, hazard ratio; BRCA, breast cancer gene; ER, oestrogen receptor; PR, progesterone receptor; HER2, human epidermal growth factor receptor 2; AC-T, doxorubicin and cyclophosphamide followed by paclitaxel; pCR, pathological complete response; ypN0, summative pathological node negativity after neoadjuvant chemotherapy; CCI, Comprehensive Complication Index.

## Discussion

There is a paucity of studies assessing the prevalence and prognostic impact of sarcopenia in patients with breast cancer receiving NAC. The results of the present study indicate that sarcopenia is not associated with baseline clinicopathological parameters, pathological response to chemotherapy, postoperative complications, or survival outcomes; however, reduced LBM is associated with a more adverse long-term oncological outcome, reflected in reduced iDFS and OS. These findings suggest that assessment of LBM may provide valuable information with respect to the overall performance status of the patient. Future studies should have a larger cohort to facilitate subgroup analyses, explore longitudinal changes in body composition, and investigate the impact of sarcopenia on toxicity relating to NAC in patients with breast cancer.

In the present study, the prevalence of sarcopenia was 12.2%, consistent with previous studies performed in Western populations^17,40^, but lower than that recently reported in a population of patients receiving NAC for breast cancer in South Korea (25.2%)^19^. This may reflect differences in body habitus and relatively lower skeletal muscle mass previously reported in Asian populations^19,41,42^. The present study also showed that sarcopenia and LBM were not associated with hormone receptor positivity or tumour molecular subtype; previous studies^43^ have similarly reported no association between skeletal muscle volume and hormone receptor status or molecular subtype characteristics. Sarcopenia was less common among patients with a higher BMI; sarcopenia has similarly been shown to be negatively correlated with BMI among patients with breast cancer^17^, although, interestingly, an increasing prevalence of obesity may be contributing to a wider increase in the syndrome of sarcopenic obesity.

Sarcopenia status was not associated with markers of response to NAC, including pCR and Sataloff tumour response. These findings are consistent with previous studies. One previous study^44^, which included 129 patients with breast cancer undergoing NAC, also found that pCR was unaffected by sarcopenia, whereas a second study^45^ found that changes in muscle mass, as indicated by pectoralis muscle area assessed using magnetic resonance imaging, did not affect the response to NAC in breast cancer. Interestingly, in the present study, greater LBM was independently associated with an increased risk of pathological nodal positivity on multivariable analysis. The reason for this finding is uncertain, but may reflect differences in disease biology among younger patients and those with premenopausal breast cancer^46^; further research is needed to explore this finding.

The results of the present study showed that sarcopenia and LBM were not associated with the occurrence or overall burden of complications for patients receiving NAC for breast cancer; there was also no association with specific complications. However, the low rate of complications in the present study cohort (15.0%), combined with the low prevalence of sarcopenia (12.2%), limited this analysis. Broyles *et al.*^47^ similarly found no association between sarcopenia status and postoperative complications following delayed abdominal free flap breast reconstruction. A separate cohort study looking at 692 patients with stage IIII breast cancer found that sarcopenia status did not affect complication rates following wide local excision or mastectomy^20^. In contrast, Kim *et al*.^48^ found that sarcopenia was associated with increased complications following deep inferior epigastric perforator flap reconstruction. That study^48^ had a larger cohort of 557 patients with a complication rate of 23.0%, whereas 46.9% of patients in the present study received breast-conserving surgery, with a lower complication rate of 15.0%. As such, although sarcopenia was not associated with increased operative morbidity in the present study, it is possible that it may be a relevant predictive factor among patients undergoing relatively higher-risk procedures, such as mastectomy with autologous reconstruction.

A key finding of the present study is that although sarcopenia status itself was not predictive with respect to long-term oncological outcome, patients with lower LBM exhibited poorer iDFS and OS, with a non-significant trend towards reduced DSS. These data suggest that a numerical increase in skeletal muscle mass is associated with improved survival, but the dichotomized definition of sarcopenia used in the present study had no prognostic value. This could be because current thresholds for the definition of sarcopenia, which were established based on cohorts with a variety of solid organ tumours, and predominantly in the metastatic setting^39^ , may not be optimal for prognostication among patients with breast cancer being treated with curative intent. Indeed, in many studies, the included patients with breast cancer are younger and predominantly female^47^, and future research should elucidate the impact that these differences may have on the prognostic value of sarcopenia in breast cancer compared with other malignancies.

The discordance between the effect of reduced LBM on OS and DSS, may indicate that the observed prognostic effect of LBM is mediated by increased non-cancer mortality. An alternative hypothesis, supported by the association between LBM and iDFS, is that the absence of an observed effect with respect to DSS may reflect the relatively lower event rate for this endpoint, reducing the power of this analysis and thus resulting in a type II error. As such, it is possible that the impact of LBM on long-term outcomes in patients receiving NAC for breast cancer may be multifactorial, mediated by both differences in performance status, and hence treatment tolerance, as well as differences in non-cancer mortality.

These findings are consistent with previous research. A large cohort study^43^ including 1460 patients with breast cancer who were being treated with curative intent found that higher skeletal muscle volume, as assessed using cross-sectional CT images, was associated with improved outcomes, including OS and recurrence-free survival. The association between low muscle mass and poor survival outcomes is consistent with the view that sarcopenia is an indicator of frailty, associated with impaired functional performance and reduced physiological reserve^49^.

The present study emphasises the clinical and prognostic value of measuring LBM. LBM could eventually be incorporated into additional clinical decision-making, including dose-adjustments for chemotherapy, although further research is needed for this to happen^12^. The prognostic value of LBM could also give it a therapeutic utility; exercise programmes have been shown to increase skeletal muscle mass and have been associated with decreased relapse and mortality rates in patients being treated for locally advanced breast cancer^50–52^.

A number of limitations must be acknowledged. First, body composition was determined by CT images obtained at diagnosis, and it is possible that changes in LBM may have occurred during NAC, as has been shown in patients with breast cancer^53^. Therefore, it is possible that preoperative assessment of body composition may result in improved predictive value for the incidence of postoperative morbidity. Future research should incorporate longitudinal assessment of body composition throughout the patient journey, in addition to assessment of muscle function and performance status. Second, given the tertiary referral nature of the centre, detailed data regarding NAC toxicity were not available for all patients, and this is suggested as an area for future research, because reduced LBM has been associated with increased chemotherapy toxicity in numerous cancer types due to the accumulation of hydrophilic compounds within the lean tissue, resulting in a reduced volume of distribution^54^. Finally, the incidence of sarcopenia in the present study was relatively low, limiting statistical power and the capacity for subgroup analysis. Notwithstanding, the present study was suitably powered to facilitate the multivariable regression analyses, in accordance with the rule of tens^55^. Future large-scale studies and meta-analyses are needed to further evaluate the impact of LBM among patients undergoing NAC for breast cancer.

## Supplementary Material

zraf128_Supplementary_Data

## Data Availability

Data will be made available upon reasonable request from the corresponding author
