# Supplementary material for: Effect of sarcopenia on treatment response and operative and oncological outcomes among patients undergoing neoadjuvant chemotherapy for breast cancer
Source: BJS Open. 2025 Nov 4;9(6):zraf128. doi: 10.1093/bjsopen/zraf128 (PMC12586920; doi:10.1093/bjsopen/zraf128)
Supplement: zraf128_Supplementary_Data [file zraf128_supplementary_data.docx]

**The effect of sarcopenia on treatment response, operative and oncologic outcomes among patients undergoing neoadjuvant chemotherapy for breast cancer**

^1^Thomas Butler, ^1^Jessie A Elliott, ^1^Matthew G Davey, ^2^Patrick M Collins, ^3^Megan McNamara, ^3^Eoin O'Malley, ^2^Micheal J Brennan, ^2^Kevin Barry, ^2^Sami Abd Elwahab, ^2^Karl Sweeney, ^2^Carmel Malone, ^2^Ray McLaughlin, ^1,2^Aoife Lowery, ^1,2^Michael J Kerin

^1^Lambe Institute for Translational Research, Department of Surgery, University of Galway, Galway, Ireland, H91 TK33

^2^University Hospital Galway, Department of Surgery, Newcastle Road, Galway, H91 YR71

^3^University Hospital Galway, Department of Radiology, Newcastle Road, Galway, H91 YR71

**Corresponding author.** Professor Michael Kerin, Lambe Institute for Translational Research, Department of Surgery, University of Galway, Galway, Ireland, H91 TK33. michael.kerin@universityofgalway.ie **ORCID ID**; https://orcid.org/0000-0003-4164-5561

**Supplementary Materials - Index**

| **Supplementary Figures and Tables** |  |
| --- | --- |
| Supplementary Table 1 | *page 2* |
| Supplementary Table 2 | *page 3* |
| Supplementary Table 3 | *page 4* |
| Supplementary Table 4 | *page 5* |
| Supplementary Table 5 | *page 6* |
| Supplementary Figure 1 | *page 7* |
| Supplementary Figure 2 | *page 7* |
| Supplementary Figure 3 | *page 8* |

**Supplementary Figures and Tables**

| **Supplementary Table 1** Association between sarcopenia and body composition | | | | | |
| --- | --- | --- | --- | --- | --- |
|  | **No sarcopenia (n=173)** | **Sarcopenia (n=24)** | ***P*** | **LBM (kg)** | ***P*** |
| **Body Anthropometry and Body Composition** |  |  |  |  |  |
| BMI category |  |  |  |  |  |
| Normal weight | 46 (31.5) | 14 (73.7) | 0.001^‡‡^ | 39.7 (4.3) | <0.001^¶^ |
| Overweight | 59 (40.4) | 5 (26.3) |  | 41.8 (4.0) |  |
| Obese | 41 (28.1) | 0 (0) |  | 45.6 (4.6) |  |
| Weight at diagnosis, kg* | 74.1 (15.1) | 63.3 (7.9) | <0.001^†^ | - | <0.001^ρ^ |
| Height, cm* | 161.7 (5.8) | 164.5 (5.0) | 0.025^†^ | - | 0.003^ρ^ |
| BMI at diagnosis* | 28.2 (5.8) | 23.2 (2.9) | <0.001^†^ | - | <0.001^ρ^ |
| BMI Preop* | 28.3 (6.1) | 22.4 (2.9) | 0.001^†^ | - | - |
| % BWL* | -0.1 (5.0) | -1.0 (6.7) | 0.556^†^ | - | 0.220^ρ^ |
| LTA, cm^2^* | 130.0 (14.1) | 96.7 (8.0) | <0.001^†^ | - | - |
| SFA, cm^2^* | 188.3 (93.3) | 141.5 (53.8) | 0.048^†^ | - | - |
| VFA, cm^2^* | 87.3 (65.3) | 50.9 (40.4) | <0.001^†^ | - | - |
| TFA, cm^2^* | 255.0 (139.9) | 181.8 (77.6) | 0.004^†^ | - | - |
| Visceral obesity | 84 (48.6) | 6 (25) | 0.030^§^ | 42.4 (5.0) [40.9 (4.5)] | 0.008^†^ |
| LBM, kg* | 42.6 (4.2) | 35.1 (2.4) | <0.001^†^ | - | - |
| SMI, cm^2^/m^2^* | 46.7 (5.4) | 35.7 (1.8) | <0.001^†^ | - | - |
| FM, kg* | 21.9 (5.9) | 18.8 (3.3) | 0.004^†^ | - | - |
| Sarcopenia | - | - | - | 35.1 (2.4) [42.6 (4.2)] | <0.001^†^ |
| Values in parentheses are percentages unless indicated otherwise, *values are mean (SD), all lean body mass values are given as mean (SD), for lean body mass, values in square parentheses denote comparison mean (SD) used to calculate P-value  SD, Standard deviation; BMI, Body Mass Index; LBM, lean body mass; LTA, Lean Tissue Area; SFA, Subcutaneous fat area; VFA, visceral fat area; TFA, Total fat area; SMI, skeletal muscle index; FM, Fat mass;  † Student independent t-test, ‡ Fisher’s exact test, § χ2 test, ¶ one-way ANOVA test, ρ Pearson’s correlation coefficient test, ‡‡ Fisher-Freeman-Halton test. | | | | | |

| **Supplementary Table 2** Association between sarcopenia and clinical characteristics | | | | | |
| --- | --- | --- | --- | --- | --- |
|  | **No sarcopenia (n=173)** | **Sarcopenia (n=24)** | ***P*** | **LBM (kg)** | ***P*** |
| **Clinical Characteristics** |  |  |  |  |  |
| Symptomatic | 160 (92.5) | 20 (83.3) | 0.135^‡^ | 41.7 (4.7) [40.7 (5.8)] | 0.397^†^ |
| Screen Detected | 12 (6.9) | 3 (12.5) | 0.401^‡^ | 40.9 (6.0) [41.6 (4.7)] | 0.575^†^ |
| Affected Breast |  |  |  |  |  |
| Right | 87 (50.3) | 12 (50) | 0.979^§^ | 41.6 (4.9) | 0.971^†^ |
| Left | 86 (49.7) | 12 (50) |  | 41.6 (4.7) |  |
| Clinical tumour stage |  |  |  |  | 0.428^¶^ |
| T0 |  |  |  | - |  |
| T1 | 20 (12.0) | 4 (17.4) | 0.810^§^ | 42.6 (5.4) |  |
| T2 | 115 (69.3) | 14 (60.9) |  | 41.4 (4.8) |  |
| T3 | 27 (16.3) | 4 (17.4) |  | 41.5 (5.2) |  |
| T4 | 4 (2.4) | 1 (4.3) |  | 39.8 (1.7) |  |
| Clinical nodal stage |  |  |  |  |  |
| N0 | 52 (33.5) | 4 (20) | 0.433^‡‡^ | 42.3 (5.2) | 0.141^†^ |
| N1-N3 | 103 (66.5) | 16 (80) |  | 41.3 (4.7) |  |
| Menopausal Status |  |  |  |  | 0.143^¶^ |
| Premenopausal | 93 (53.8) | 15 (62.5) | 0.830^‡‡^ | 42.1 (4.8) |  |
| Perimenopausal | 12 (6.9) | 1 (4.2) |  | 42.0 (5.5) |  |
| Postmenopausal | 68 (39.3) | 8 (33.3) |  | 40.9 (4.6) |  |
| Menopausal status at presentation** | - | - | - | 42.1 (4.9) [40.9 (4.6)] | 0.048^†^ |
| Age at Diagnosis, years* | 49.7 (11.0) | 45.9 (9.9) | 0.108^†^ | - | 0.067^ρ^ |
| Clinical Tumour Size in mm* | 38.9 (18.4) | 38.7 (21.7) | 0.960^†^ | - | 0.947^ρ^ |
| New NPI |  |  |  |  |  |
| NPI 1 | 2 (1.4) | 0 (0) | 0.887^‡‡^ | 42.3 (6.4) | 0.126^¶^ |
| NPI 2 | 3 (2.1) | 0 (0) |  | 47.4 (1.9) |  |
| NPI 3 | 90 (64.3) | 11 (61.1) |  | 41.9 (4.9) |  |
| NPI 4 | 45 (32.1) | 7 (38.9) |  | 41.0 (4.8) |  |
| Values in parentheses are percentages unless indicated otherwise, *values are mean (SD) **analysed as post-menopausal vs pre or peri-menopausal, all lean body mass values are given as mean (SD), for lean body mass, values in square parentheses denote comparison mean (SD) used to calculate P-value  SD, Standard deviation; NPI, Nottingham Prognostic Index; LBM, lean body mass  † Student independent t-test, ‡ Fisher’s exact test, § χ2 test, ¶ one-way ANOVA test,  ^ρ^ Pearson’s correlation coefficient test, ‡‡ Fisher-Freeman-Halton test. | | | | | |

| **Supplementary Table 3** Association between sarcopenia and pathological characteristics | | | | | |
| --- | --- | --- | --- | --- | --- |
|  | **No sarcopenia (n=173)** | **Sarcopenia (n=24)** | ***P*** | **LBM (kg)** | ***P*** |
| **Pathological Characteristics** |  |  |  |  |  |
| Tumour grade |  |  |  |  |  |
| Grade 1 | 3 (1.8) | 0 (0) | 0.882^‡‡^ | 44.6 (4.0) | 0.215^¶^ |
| Grade 2 | 85 (49.7) | 11 (47.8) |  | 41.8 (5.0) |  |
| Grade 3 | 83 (48.5) | 12 (52.2) |  | 41.3 (4.6) |  |
| Pathological Tumour Stage |  |  |  |  |  |
| T0 | 33 (20.8) | 2 (10) | 0.083^‡‡^ | 41.8 (4.7) | 0.549^¶^ |
| T1 | 36 (22.6) | 10 (50) |  | 41.6 (4.9) |  |
| T2 | 52 (32.7) | 3 (15) |  | 41.1 (4.5) |  |
| T3 | 33 (20.8) | 4 (20) |  | 42.6 (5.5) |  |
| T4 | 5 (3.1) | 1 (5) |  | 40.5 (5.0) |  |
| Pathological Nodal Stage |  |  | - |  |  |
| N0 | 67 (47.2) | 8 (44.4) | 0.945^‡‡^ | 41.7 (4.7) | 0.821^¶^ |
| N1 | 40 (28.2) | 6 (33.3) |  | 41.9 (5.2) |  |
| N2 | 21 (14.8) | 2 (11.1) |  | 41.4 (4.8) |  |
| N3 | 14 (9.9) | 2 (11.1) |  | 40.7 (5.4) |  |
| Histological Subtype |  |  |  |  |  |
| Ductal | 127 (73.4) | 19 (79.2) | 0.933^‡‡^ | 41.5 (4.8) | 0.677^¶^ |
| Lobular | 14 (8.1) | 1 (4.2) |  | 42.4 (5.4) |  |
| Other | 32 (18.5) | 4 (16.7) |  | 41.7 (4.4) |  |
| Inflammatory | 14 (20.6) | 3 (30) | 0.682^‡^ | 39.9 (4.1) [41.4 (5.0)] | 0.158^†^ |
| Multifocal | 21 (12.1) | 3 (12.5) | 1.000^‡^ | 42.2 (4.6) [41.5 (4.8)] | 0.501^†^ |
| ER | 110 (63.6) | 14 (58.3) | 0.618^§^ | 41.4 (4.9) [41.8 (4.7)] | 0.546^†^ |
| PR | 93 (53.8) | 14 (58.3) | 0.673^§^ | 41.4 (4.8) [41.8 (4.8)] | 0.561^†^ |
| HER-2 Positivity | 48 (27.7) | 9 (37.5) | 0.323^§^ | 41.3 (4.6) [41.7 (4.9)] | 0.548^†^ |
| Breast Cancer receptor type |  |  |  |  |  |
| LA | 85 (49.1) | 11 (45.8) | 0.746^‡‡^ | 41.5 (4.7) | 0.733^¶^ |
| LB | 30 (17.3) | 5 (20.8) |  | 41.1 (5.0) |  |
| HER2 | 19 (11.0) | 4 (16.7) |  | 41.6 (3.7) |  |
| Basal | 39 (22.5) | 4 (16.7) |  | 42.1 (5.2) |  |
| ER Score* | 4.6 (3.6) | 4.2 (3.6) | 0.593^†^ | - | 0.508^ρ^ |
| PR Score* | 3.4 (3.3) | 3.0 (3.2) | 0.534^†^ | - | 0.696^ρ^ |
| Ki67%* | 27.8 (21.4) | 28.0 (11.4) | 0.980^†^ | - | 0.671^ρ^ |
| Background DCIS | 97 (71.9) | 16 (94.1) | 0.073‡ | 41.6 (5.0) [41.5 (4.3)] | 0.832^†^ |
| Grade of DCIS |  |  |  |  |  |
| Grade 1 | 2 (2.4) | 0 (0) | 0.339^‡‡^ | - | - |
| Grade 2 | 20 (23.8) | 1 (6.7) |  | - | - |
| Grade 3 | 62 (73.8) | 14 (93.3) |  | - | - |
| BRCA 1 | 3 (1.7) | 1 (4.2) | 0.408^‡^ | 38.7 (4.9) [41.7 (4.8)] | 0.134^†^ |
| BRCA 2 | 2 (1.2) | 0 (0) | 1.000^‡^ | 41.4 (7.7) [41.6 (4.8)] | 0.960^†^ |
| Triple negative | 23 (21.3) | 1 (7.1) | 0.299^‡^ | 41.3 (4.5) [41.7 (4.8)] | 0.657^†^ |
| Tumour Bed size, mm* | 36.5 (24.5) | 28.7 (15.6) | 0.458^†^ | - | 0.797^ρ^ |
| Values in parentheses are percentages unless indicated otherwise, *values are mean (SD), all lean body mass values are given as mean (SD), for lean body mass, values in square parentheses denote comparison mean (SD) used to calculate P-value  SD, Standard deviation; LBM, lean body mass; ER, Oestrogen receptor; PR, Progesterone receptor; HER2, human epidermal growth factor receptor 2; BRCA, breast cancer gene; LA, luminal A; LB Luminal B; DCIS, ductal carcinoma *in situ,* LCIS, lobular carcinoma *in situ;*  † Student independent t-test, ‡ Fisher’s exact test, § χ2 test, ¶ one-way ANOVA test,  ^ρ^ Pearson’s correlation coefficient test, ‡‡ Fisher-Freeman-Halton test. | | | | | |

| **Supplementary Table 4** Association between sarcopenia and treatment characteristics | | | |  |  |
| --- | --- | --- | --- | --- | --- |
|  | **No sarcopenia (n=173)** | **Sarcopenia (n=24)** | ***P*** | **LBM (kg)** | ***P*** |
|  |  |  |  |  |  |
| **Treatment Characteristics** |  |  |  |  |  |
| NAC regimen |  |  |  |  |  |
| AC-T | 127 (74.3) | 15 (62.5) | 0.225^§^ | 41.1 (5.7) | 0.775^κ^ |
| Other | 44 (25.7) | 9 (37.5) |  | 40.9 (5.5) |  |
| Neoadjuvant trastuzumab |  |  |  |  |  |
| No neoadjuvant trastuzumab | 127 (74.3) | 15 (62.5) | 0.297^‡‡^ | 41.6 (4.9) | 0.635^†^ |
| Neoadjuvant trastuzumab | 39 (22.8) | 9 (37.5) |  | 41.3 (4.3) |  |
| Neoadjuvant trastuzumab + lapatinib | 5 (2.9) | 0 (0) |  | - | - |
| Sentinel node procedure before NAC | 23 (13.3) | 2 (8.3) | 0.745^‡^ | 42.6 (4.8) [41.4 (4.8)] | 0.180^†^ |
| Surgery Performed |  |  |  |  |  |
| WLE | 83 (48.0) | 12 (50) | 0.853^§^ | 42.0 (4.8) | 0.238^†^ |
| Mastectomy | 90 (52.0) | 12 (50) |  | 41.3 (4.8) |  |
| Axillary Procedure |  |  |  |  |  |
| SLNB | 43 (24.9) | 5 (20.8) | 0.667^§^ | 41.9 (5.1) | 0.595^†^ |
| ALND | 130 (75.1) | 19 (79.2) |  | 41.5 (4.7) |  |
| R0 | 142 (98.6) | 18 (100) | 1.000^‡^ | 41.6 (4.8) | 0.656^†^ |
| Reoperation for completion mastectomy to achieve R0 | 8 (5.1) | 0 (0) | 0.598^‡^ | 43.2 (5.8) [41.5 (4.8)] | 0.282^†^ |
| Reconstruction Performed | 52 (30.1) | 11 (45.8) | 0.120^§^ | 41.7 (4.9) [41.6 (4.8)] | 0.885^†^ |
| Adjuvant Chemotherapy | - | - | - | 39.0 (6.1) [41.7 (4.7)] | 0.056^†^ |
| Adjuvant hormonal treatment | 54 (31.2) | 8 (33.3) | 0.834^§^ | 41.8 (4.6) [41.5 (4.9)] | 0.605^†^ |
| Adjuvant radiotherapy | 158 (91.9) | 23 (95.8) | 0.699^‡^ | 41.6 (4.7) [41.6 (5.9)] | 0.956^†^ |
| Boosts | 78 (45.1) | 15 (62.5) | 0.109^§^ | - | - |
| Cycles complete* | 4.5 (1.2) | 4.5 (1.1) | 0.883^†^ | - | 0.695^ρ^ |
| Dose, Gy* | 48.9 (3.3) | 50.1 (0.2) | <0.001^†^ | - | 0.429^ρ^ |
| Number of fractions* | 24.8 (3.9) | 25.8 (1.4) | 0.2^†^ | - | 0.608^ρ^ |
|  |  |  |  |  |  |
| Values in parentheses are percentages unless indicated otherwise, *values are mean (SD), all lean body mass values are given as mean (SD), for lean body mass, values in square parentheses denote comparison mean (SD) used to calculate P-value, where Kruskal-Wallis test was performed, median (interquartile range) is reported  SD, Standard deviation; LBM, lean body mass; AC-T, doxorubicin and cyclophosphamide followed by paclitaxel; T, paclitaxel; AC, doxorubicin and cyclophosphamide; TAC, docetaxel, doxorubicin and cyclophosphamide; NAC, neoadjuvant chemotherapy; WLE, wide local excision; SLNB, sentinel lymph node biopsy; ALND, axillary lymph node dissection;  † Student independent t-test, ‡ Fisher’s exact test, § χ2 test, ¶one-way ANOVA test, ^ρ^ Pearson’s correlation coefficient test, ‡‡ Fisher-Freeman-Halton test, κ Kruskal-Wallis test. | | | | | |

| **Supplementary Table 5** Multivariable analysis of factors associated with postoperative outcomes | | | | | | |
| --- | --- | --- | --- | --- | --- | --- |
|  | **CCI** | | **LOS** | | **Any Complication** | |
|  | ***P*-value** | **B (SE)** | ***P*-value** | **B (SE)** | ***P*-value** | **OR (95% CI)** |
| **Clinicopathological characteristics** |  |  |  |  |  |  |
| Age, years | 0.519 | - | 0.580 | - | 0.286 | - |
| Menopausal status | 0.709 | - | 0.728 | - | 0.429 | - |
| Tumour grade, G3 vs G1-2 | 0.283 | - | 0.064 | - | 0.467 | - |
| Histological subtype* | 0.011 | 2.94 (1.14) | 0.510 | - | 0.284 | - |
| Clinical tumour size, mm | 0.590 | - | 0.742 | - | 0.196 | - |
| Clinical Tumour stage* | 0.179 | - | 0.622 | - | 0.499 | - |
| Clinical Nodal stage, cN1-3 vs cN0 | 0.357 | - | 0.350 | - | 0.782 | - |
| ER status | 0.259 | - | 0.559 | - | 0.270 | - |
| ER Allred score | 0.202 | - | 0.957 | - | 0.268 | - |
| PR status | 0.471 | - | 0.003 | -2.50 (0.84) | 0.772 | - |
| PR Allred score | 0.610 | - | 0.119 | - | 0.676 | - |
| Screen Detected | 0.656 | - | 0.540 | - | 0.990 | - |
| Pathological Nodal stage | 0.333 | - | 0.997 | - | - | - |
| Tumour multifocality | 0.001 | 7.42 (2.22) | 0.060 | - | 0.006 | 4.44 (1.54-12.83) |
| HER-2 Positivity | 0.290 | - | 0.893 | - | 0.474 | - |
| Breast Cancer receptor type | 0.134 | - | 0.032 | -0.75 (0.35) | 0.755 | - |
| BRCA Association | 0.136 | - | 0.200 | - | 0.120 | - |
|  |  |  |  |  |  |  |
| **Treatment characteristics** |  |  |  |  |  |  |
| Surgery Performed | 0.519 | - | <0.001 | 2.36 (0.56) | 0.635 | - |
| Axillary Procedure Performed | 0.154 | - | <0.001 | 2.38 (0.63) | 0.434 | - |
| Reconstruction Performed | 0.077 | - | 0.146 | - | 0.633 | - |
| Neoadjuvant chemotherapy regimen - Other vs AC-T | 0.286 | - | 0.815 | - | 0.354 | - |
| Neoadjuvant HER2-directed treatment | 0.072 | - | 0.179 | - | 0.042 | 0.30 (0.10-0.96) |
| Cycles complete | - | - | - | - | 0.766 | - |
|  |  |  |  |  |  |  |
| **Treatment response characteristics** |  |  |  |  |  |  |
| pCR | 0.805 | - | 0.464 | - | - | - |
| Downstaging | 0.094 | - | 0.386 | - | - | - |
| Pathologic tumour size, mm | 0.908 | - | 0.493 | - | - | - |
| Sataloff Tumour Response, A-B vs C-D | 0.689 | - | 0.398 | - | - | - |
| Post-NAC size | 0.861 | - | 0.806 | - | - | - |
|  |  |  |  |  |  |  |
| **Body Composition** |  |  |  |  |  |  |
| Sarcopenia | 0.242 | - | 0.716 |  | 0.365 | - |
| LBM, kg | 0.161 | - | 0.062 | - | 0.352 | - |
| OR, odds ratio; CI, confidence interval; SE, standard error; LBM, lean body mass; CCI, comprehensive complication index; LOS, length of stay; pCR, pathologic complete response; ER, Oestrogen receptor; PR, Progesterone receptor; HER2, human epidermal growth factor receptor 2; BRCA, breast cancer gene; AC-T, doxorubicin and cyclophosphamide followed by paclitaxel; NAC, neoadjuvant chemotherapy  *Analysed as a categorical variable, category P-values and Hazard Ratios not significant on multivariable linear and Cox proportional hazards regression | | | | | | |


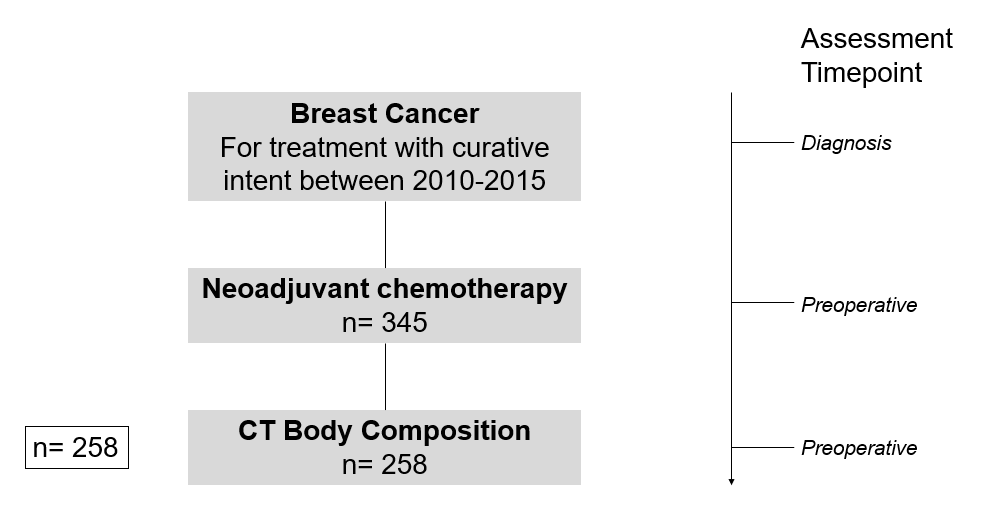


**Supplementary Figure 1.** All patients with breast cancer who underwent neoadjuvant chemotherapy with curative intent between 2010-2015 were assessed for inclusion. Patients without a CT scan suitable for analysis and with non-progression to surgery after chemotherapy were excluded.


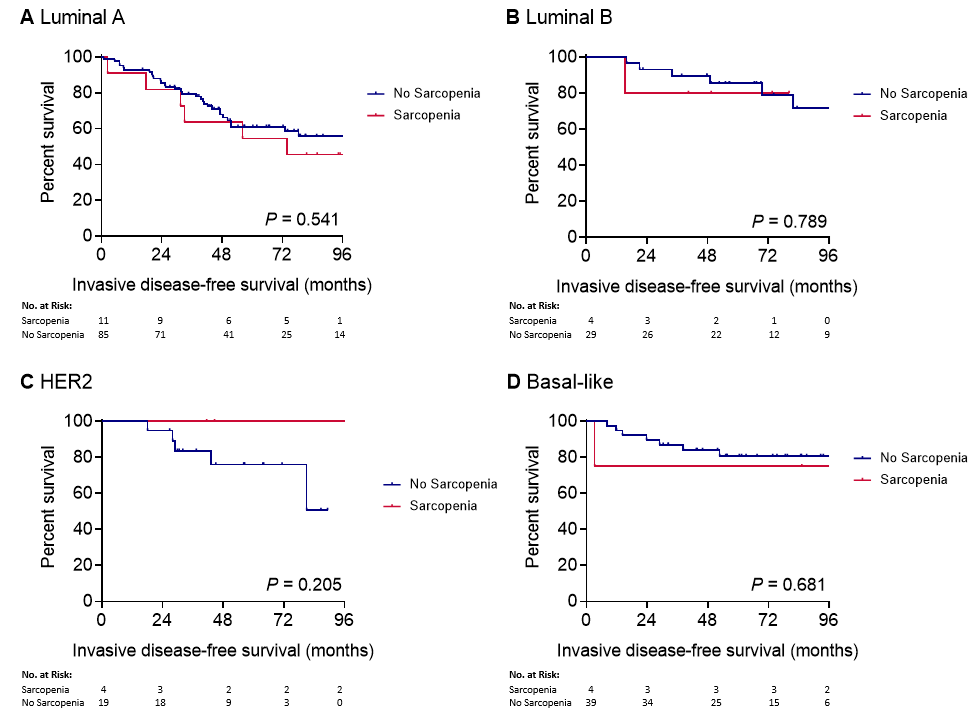


**Supplementary Figure 2.** Sarcopenia and survival outcome according to molecular subtype

**Supplementary Figure 3.** Graphs to demonstrate survival outcomes and LBM quartiles

OS, overall survival; iDFS, invasive disease-free survival; DSS, disease specific survival; LBM, lean body mass
